# Nanomechanical Characterization of Canine Femur Bone for Strain Rate Sensitivity in the Quasistatic Range under Dry versus Wet Conditions

**DOI:** 10.1155/2012/415230

**Published:** 2012-12-25

**Authors:** Kun-Lin Lee, Marta Baldassarri, Nikhil Gupta, Dinesh Pinisetty, Malvin N. Janal, Nick Tovar, Paulo G. Coelho

**Affiliations:** ^1^Composite Materials and Mechanics Laboratory, Mechanical and Aerospace Engineering Department, Polytechnic Institute of New York University, Brooklyn, NY 11201, USA; ^2^Department of Biomaterials and Biomimetics, College of Dentistry, New York University, 345 24th Street 813a, New York, NY 10010, USA; ^3^Department of Epidemiology, New York University, NY 10010, USA; ^4^Department of Periodontology and Implant Dentistry, College of Dentistry, New York University, 345 24th Street 813a, New York, NY 10010, USA

## Abstract

As a strain rate-dependent material, bone has a different mechanical response to various loads. Our aim was to evaluate the effect of water and different loading/unloading rates on the nanomechanical properties of canine femur cortical bone. Six cross-sections were cut from the diaphysis of six dog femurs and were nanoindented in their cortical area. Both dry and wet conditions were taken into account for three quasistatic trapezoid profiles with a maximum force of 2000 **μ**N (holding time = 30 s) at loading/unloading rates of 10, 100, and 1000 **μ**N/s, respectively. For each specimen, 254 ± 9 (mean ± SD) indentations were performed under different loading conditions. Significant differences were found for the elastic modulus and hardness between wet and dry conditions (*P* < 0.001). No influence of the loading/unloading rates was observed between groups except for the elastic modulus measured at 1000 **μ**N/s rate under dry conditions (*P* < 0.001) and for the hardness measured at a rate of 10 **μ**N/s under wet conditions (*P* < 0.001). Therefore, for a quasistatic test with peak load of 2000 **μ**N held for 30 s, it is recommended to nanoindent under wet conditions at a loading/unloading rate of 100–1000 **μ**N/s, so the reduced creep effect allows for a more accurate computation of mechanical properties.

## 1. Introduction

Bone is characterized by a complex hierarchical composite structure, comprising of mineral and organic matrix, 90% of which is collagen type I [1, 2]. The hard mineral, whose mechanical behavior is similar to that of a ceramic material, determines the strength and the stiffness of the tissue and does not affect its strain-rate sensitivity [3–5]. On the other hand, collagen, as a viscoelastic material, contributes to the rate-dependent fracture toughness of bone [1, 3, 6–9]. The combination of mineral and organic phases determines the unique mechanical properties of bone [5]. Under loading, slipping of collagen fibers is reduced by higher resistance at the collagen-mineral interface relative to the organic phase alone [5]. 

Bone hydration significantly affects the mechanical behavior of the tissue [2]. Water contributes to lower stiffness, elastic modulus, hardness, and higher recoverable strain from creep [10–14]. Water is found in the vascular canals, lacunae, and canaliculi. Due to its polarity, it binds with hydrophilic groups of collagen proteins and charged groups of bone mineral [2, 15]. The interaction of water with these components, occurring at the nanomicrostructural level, affects the mechanical properties of bone [16]. A previous study has quantified the effect of water on the mechanical response of human bone [17]. In this study the modulus and hardness of dry bone were found to be about 23 and 57%, respectively, higher than that of wet bone.

As a viscoelastic tissue, bone is a strain-rate dependent material. When it is subjected to different loading rates, as occurs in the body, bone exhibits different stress and strain values. Various studies have shown that the mechanical properties of bone are slightly affected by the loading rate applied during the test [17–20].

Several techniques have been used to perform mechanical tests on bone. Micro- and nanoindentation methods have been used to measure the mechanical properties of bone determined by its nanomicroscale hierarchical structure. Microindentation allows hardness but not elastic modulus measurements, whereas with nanoindentation both hardness and elastic modulus values can be obtained. Furthermore, with nanoindentation small-sized specimens can be used to obtain many observations [21]. As this technique is now widely used to mechanically characterize bone [21–26], validation of loading test parameters, such as hydration, is needed [21]. A previous study by Hoffler et al., 2005 [17] has focused on the influence of specimen preparation/testing condition, indentation depth, repetitive loading, time delay, and displacement rate on the nanomechanical properties of human bone. However, many investigations focus on the mechanical properties of other tissues, such as dogs. Therefore, it is critical to assess the nanomechanical properties of canine bone. In our study, we aimed to evaluate nanoindentation testing parameters by assessing the effect of hydration and loading/unloading rate on the elastic modulus and hardness of canine bone. Three quasistatic test profiles with different loading/unloading rates (10, 100, and 1000 *μ*N/s) were developed to assess the influence of loading rate on the mechanical response of the tissue.

## 2. Materials and Methods

### 2.1. Specimen Preparation

Six canine femurs were obtained postmortem from six dogs approximately eighteen months old, soaked in 70% ethanol, and frozen at −20°C, which has been shown to have no effect on the mechanical properties of bone if adequately rehydrated [12]. One cross-sectional segment, around 10 mm in length, was cut from the diaphysis of each femur using a precision diamond saw (Isomet 2000, Buelher, Lake Bluff, IL, USA) under continuous water irrigation and embedded using polymethyl-methacrylate (PMMA) resin (Fisher Scientific, Waltham, MA, USA). Previous investigations have shown that the embedding procedure has no significant influence on the mechanical properties of bone measured with nanoindentation [17]. One specimen, with a thickness of 30 *μ*m along the longitudinal axis, was obtained from each bone segment by performing two parallel cuts in the cross-sectional direction. Specimens were glued to acrylic plates with acrylate-based cement (Fisher Scientific, Waltham, MA, USA). After a setting time of 24 h, grinding (400 to 2400 grit SiC abrasive paper), and polishing (diamond suspensions of 9 to 1 *μ*m particle size) (Buehler, Lake bluff, IL, USA) were performed and a final thickness of approximately 20 *μ*m was reached. All specimens were frozen at −20°C until two hours prior to mechanical testing [12].

### 2.2. Nanoindentation Testing

In total, 1466 indentations were performed, with an average of 254 ± 9 (average ± standard deviation) indentations per specimen. A nanoindenter (Hysitron, Minneapolis, MN, USA) equipped with a Berkovich diamond three-sided pyramid probe was used. Indentations in the same specimen were performed with a distance of at least 10 *μ*m from each other and from bone boundaries, so that no interactions between them affected the mechanical results [27]. For each specimen, indentations were carried out under both dry and then wet conditions. For testing under wet conditions, after bringing the specimen to room temperature, hydration was performed by adding distilled water into a wax chamber created around the bone perimeter two hours before testing. Attention was paid during the tests to assure that water was always present in the chamber. For both conditions (wet, dry), each specimen was loaded with a quasistatic profile involving a peak load of 2000 *μ*N at a loading rate of either 10, 100, or 1000 *μ*N/s, held constant for a period of time of 30 s and an unloading rate of 10, 100, and 1000 *μ*N/s, respectively. A holding time of 30 s was chosen to allow the displacement of the viscoelastic bone to reach a more steady response, so the Oliver-Pharr model could be applied to the data obtained [28, 29]. Indentations performed under different conditions and/or loading rates were located in the same regions within each specimen but ranged from the inner to the outer cortical shell in an attempt to avoid structural heterogeneity effects on the results [17]. 

**Figure 1:**
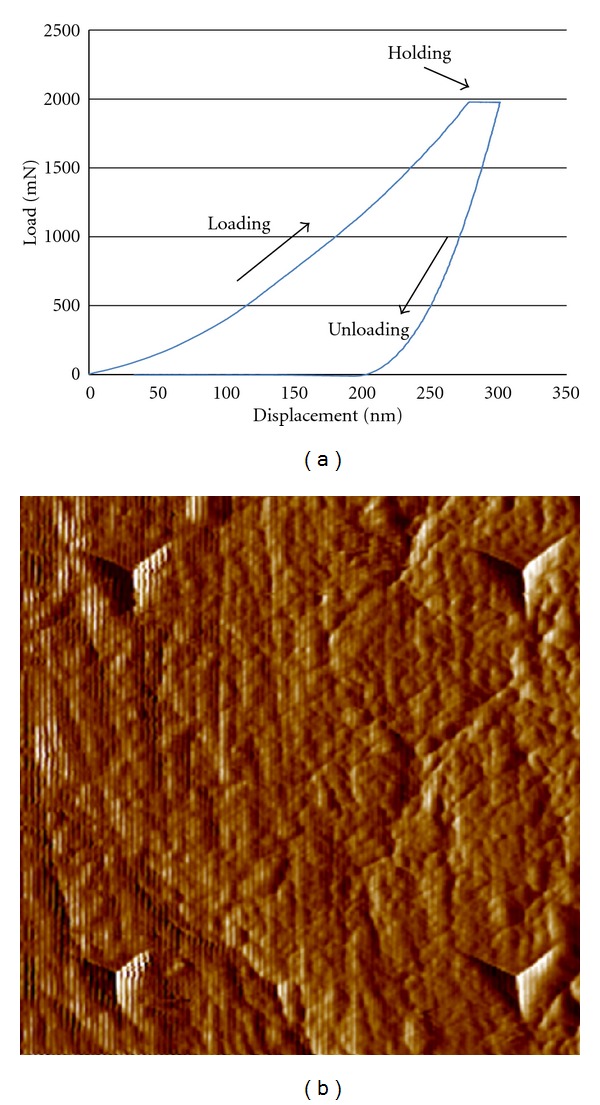
(a) A representative load-displacement graph obtained from nanoindentation testing. (b) Four indents seen on the surface of a bone specimen.

From each indentation, a load-displacement curve was obtained and residual modulus *E*_*r*_ (GPa) and hardness (GPa) were calculated [28]. The elastic modulus *E* (GPa) was computed from the following:

(1) $$\begin{array}{c} \frac{1}{E_{r}}=\frac{1-\nu^{2}}{E}+\frac{1-\nu_{i}^{2}}{E_{i}}, \end{array}$$

where *ν*(0.3) is the Poisson's ratio of cortical femur bone [22], and *E*_*i*_ (1141 GPa) and *ν*_*i*_ (0.07) are the elastic modulus and Poisson's ratio of the indenter [22]. A representative set of load displacement curve (loading-holding-unloading) and indents on the specimen are shown in Figure 1.

### 2.3. Statistical Analysis

A statistical software (SPSS, IBM, Armonk, NY, USA) was used for analyzing the data. Initial normality check was performed and data transformation to ranks executed, allowing statistical inferences by a general linear mixed model considering repeated measures within each sample. Statistical significance was set to *α* = 0.05. Preliminary statistical analysis showed no effect of animal (considered the statistical unit in the present study, *n* = 6) in each dependent variable (rank elastic modulus and rank hardness) considering both testing condition and loading rate. The influence of both testing condition (wet, dry) and loading rate (10, 100 and due to the 1000 *μ*N/s) on rank elastic modulus and hardness was assessed.

## 3. Results

**Figure 2:**
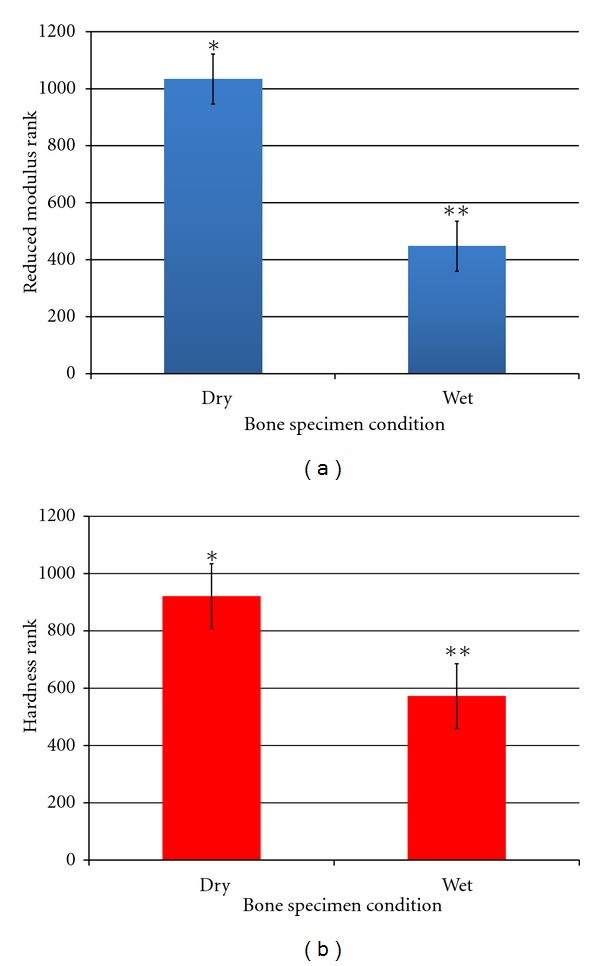
(a) Elastic modulus and (b) hardness ranks averaged over six specimens for each condition (wet, dry). Bars represent 95% confidence intervals. Different asterisks number show significant differences between groups (*P* < 0.001).

**Figure 3:**
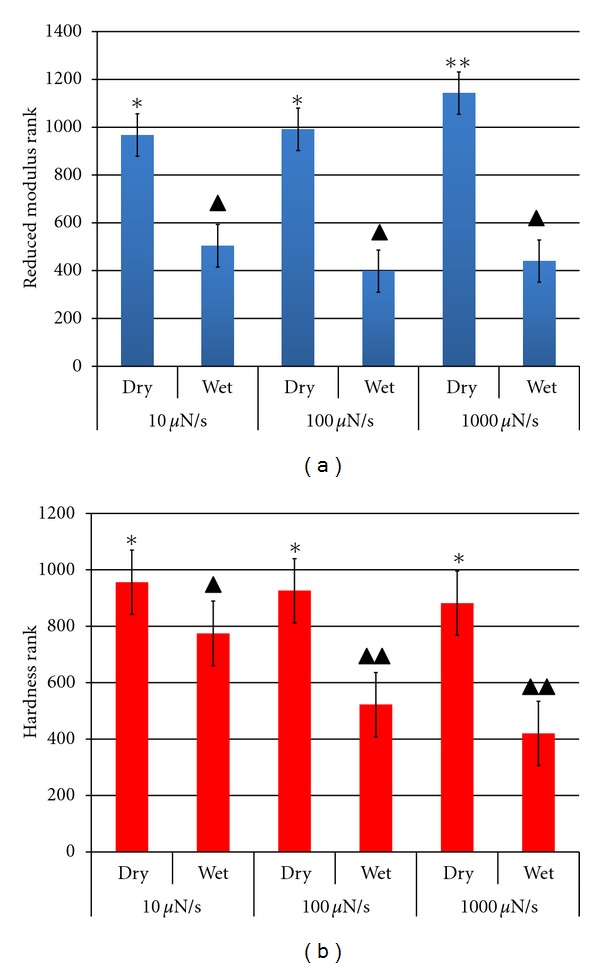
(a) Elastic modulus and (b) hardness ranks averaged over six specimens for each condition (wet, dry) and loading/unloading rate (10, 100, and 1000 *μ*N/s). Bars represent 95% confidence intervals. Different asterisks number/color show significant differences between groups (*P* < 0.001).

For each testing condition (wet, dry) and loading rate (10, 100, and 1000 *μ*N/s), elastic modulus and hardness values of the six specimens were averaged (Table 1). When ranked data were analyzed, the testing condition significantly affected both the elastic modulus and the hardness of bone (*P* < 0.001) (Figures 2(a) and 2(b)). No difference was found for the elastic modulus when the effect of different loading rates was investigated (Figure 3(a)). However, hardness was significantly higher when a loading rate of 10 *μ*N/s was involved relative to the other two (*P* < 0.001); no difference was shown when tests were performed at 100 and 1000 *μ*N/s loading rates (Figure 3(b)). 

When the combination of the effect of water and loading rates was taken into account, the elastic modulus in wet specimens showed no dependence on the loading rate, while significantly higher values were found for dry specimens using 1000 *μ*N/s compared to 100 and 10 *μ*N/s (*P* < 0.001). No difference was observed when tests were performed at 100 and 10 *μ*N/s loading rates (Figure 3(a)). The hardness of wet specimens was significantly higher at a loading rate of 10 *μ*N/s relative to 100 *μ*N/s and 1000 *μ*N/s (*P* < 0.001), but no dependence on loading rates was found for the hardness of dry specimens (Figure 3(b)). 

## 4. Discussion

This study investigated the influence of water and loading rates on the elastic modulus and hardness of canine cortical bone measured with nanoindentation. For each testing condition and/or loading rate, the variation of the elastic modulus and hardness averaged among six dog bones tested specimens was likely due to the anisotropic properties of the tissue and variability found within bone samples due to its heterogeneous structure [5]. To the best our knowledge, no previous work has been carried out on the mechanical properties of canine femur cortical bone measured with nanoindentation. Therefore, no direct comparison with previous results could be performed. 

Tissue hydration significantly affected the mechanical properties of the tissue, as previously observed for human femur cortical bone [17]. In that study, dry tissue showed elastic modulus and hardness 22.6% and 56.9%, respectively, higher than those measured under wet conditions. Differently, in this study, dry bone had elastic modulus and hardness, respectively, 65% and 18% higher than those measured in wet tissue. 

Under dry conditions, no interaction of water with collagen and mineral is present. Without water, collagen fibrils might stiffen and contract longitudinally compressing the mineral phase, leading to a higher strength of bone [2]. A change of the size of mineral crystals after loss of water was also observed by LeGeros et al., 1978 [30]. For hydrated bone, the viscoelastic behavior of collagen has been shown to be significantly affected, particularly for higher loading rates [31, 32]. In the current study, the influence of hydration on the elastic modulus and hardness was more pronounced at higher loading rate (1000 *μ*N/s) relative to those at lower rates (10, 100 *μ*N/s), suggesting that at higher loading rate, wet versus dry conditions affect the mechanical response of the tissue more than at lower loading rates. The critical influence of hydration was emphasized also in a previous work in which water was hydraulically pushed out of voids in bone dynamically tested under high rate loading [3].

For higher loading rates, higher modulus and hardness are expected [17–19, 33]. However, only in a few cases the mechanical response of bone was affected by different rates, that is, the creep behavior of the tissue was not influencing the elastic modulus and hardness computation. This result was possibly due to the holding time (30 s) set for the maximum force along with the unloading rate values. For quasistatic tests performed on time-dependent specimens, as in our case, the effect of creep on the elastic modulus and hardness computation can be reduced if the tissue has sufficient time to complete its time-dependent deformation and if a relatively high unloading rate is applied. Furthermore, ISO 14577-4 suggests reducing the creep effect of the material when it is nanoindented with quasistatic profiles to allow the Oliver-Pharr method to be applied for the elastic modulus and hardness computation [28, 29]. Therefore, since hardness measured in wet bone at a loading/unloading rate of 10 *μ*N/s was significantly different from the ones measured at 100 and 1000 *μ*N/s, at the lowest rate the creep effect had not been reduced enough for the bone to be loading/unloading independent of viscoelastic effects. 

## 5. Conclusion

This study shows that the elastic modulus and hardness of canine cortical bone measured under quasistatic testing with the nanoindentation technique are strongly dependent on the environmental condition (i.e., dry versus wet). Therefore, for a quasistatic test with peak load of 2000 *μ*N held for 30 s, it is recommended to nanoindent under wet conditions at a loading/unloading rate of 100–1000 *μ*N/s, so the reduced creep effect allows for a more accurate computation of elastic modulus and hardness.

## Figures and Tables

**Table 1:** Elastic modulus (GPa) and hardness (GPa) values averaged over six specimens for each condition (wet, dry) and/or loading/unloading rate (10, 100, and 1000 *μ*N/s) (mean ± standard deviation).

Condition	Loading rate (*μ*N/s)	Elastic modulus (GPa)	Hardness (GPa)
Dry	1000	24.5 ± 0.9	0.9 ± 0.1
Dry	100	22.6 ± 5.4	0.9 ± 0.2
Dry	10	22.2 ± 5.6	1 ± 0.3

Wet	1000	14 ± 4.5	0.6 ± 0.3
Wet	100	13 ± 4.7	0.67 ± 0.4
Wet	10	15 ± 5.9	1.1 ± 1
